# Heterologous SARS‐CoV‐2 IgA neutralising antibody responses in convalescent plasma

**DOI:** 10.1002/cti2.1424

**Published:** 2022-10-23

**Authors:** Samantha K Davis, Kevin John Selva, Ester Lopez, Ebene R Haycroft, Wen Shi Lee, Adam K Wheatley, Jennifer A Juno, Amy Adair, Phillip Pymm, Samuel J Redmond, Nicholas A Gherardin, Dale I Godfrey, Wai‐Hong Tham, Stephen J Kent, Amy W Chung

**Affiliations:** ^1^ Department of Microbiology and Immunology The Peter Doherty Institute for Infection and Immunity University of Melbourne Melbourne VIC Australia; ^2^ The Walter and Eliza Hall Institute of Medical Research Melbourne VIC Australia; ^3^ Melbourne Sexual Health Centre and Department of Infectious Diseases Alfred Hospital and Central Clinical School Monash University Melbourne VIC Australia

**Keywords:** ACE2 inhibition, Fc function, IgA, neutralisation, RBD, SARS‐CoV‐2

## Abstract

**Objectives:**

Following infection with SARS‐CoV‐2, virus‐specific antibodies are generated, which can both neutralise virions and clear infection via Fc effector functions. The importance of IgG antibodies for protection and control of SARS‐CoV‐2 has been extensively reported. By comparison, other antibody isotypes including IgA have been poorly characterised.

**Methods:**

Here, we characterised plasma IgA from 41 early convalescent COVID‐19 subjects for neutralisation and Fc effector functions.

**Results:**

Convalescent plasma IgA from > 60% of the cohort had the capacity to inhibit the interaction between wild‐type RBD and ACE2. Furthermore, a third of the cohort induced stronger IgA‐mediated ACE2 inhibition than matched IgG when tested at equivalent concentrations. Plasma IgA and IgG from this cohort broadly recognised similar RBD epitopes and had similar capacities to inhibit ACE2 from binding to 22 of the 23 prevalent RBD mutations assessed. However, plasma IgA was largely incapable of mediating antibody‐dependent phagocytosis in comparison with plasma IgG.

**Conclusion:**

Overall, convalescent plasma IgA contributed to the neutralising antibody response of wild‐type SARS‐CoV‐2 RBD and various RBD mutations. However, this response displayed large heterogeneity and was less potent than IgG.

## Graphical abstract

Following infection with SARS‐CoV‐2, virus‐specific antibodies are generated, which can both neutralise virions and clear infection via Fc effector functions. Here, we demonstrate that convalescent plasma IgA was largely incapable of mediating antibody‐dependent phagocytosis in comparison with plasma IgG. However, IgA from > 60% of the cohort had the capacity to neutralise SARS‐CoV2 wild‐type and numerous RBD variants, although this response was heterogeneous and less potent than IgG.

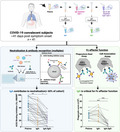

## Introduction

Severe acute respiratory syndrome coronavirus 2 (SARS‐CoV‐2), the causative agent of coronavirus disease 2019 (COVID‐19), has infected millions of people and caused over 6 million deaths globally since its discovery. The SARS‐CoV‐2 trimeric spike protein consists of two domains: spike 1 (S1) and spike 2 (S2).^1^ The receptor‐binding domain (RBD) within the S1 engages with angiotensin‐converting enzyme 2 (ACE2) on human cells contributing to infection.^1^ Antibodies generated towards RBD following infection or vaccination can block engagement with ACE2 and neutralise SARS‐CoV‐2.^2^, ^3^, ^4^ Mutations within the RBD can generate strains that have improved transmissibility and can escape antibody immunity induced by vaccination or infection, thus potentially becoming variants of concern (VOC).^5^, ^6^, ^7^ Importantly, both neutralising and non‐neutralising antibodies engage fragment crystallisable (Fc) receptors on immune cells (e.g. monocytes) to activate Fc effector functions and clear infection.^8^, ^9^, ^10^, ^11^ This polyfunctional antibody response assists in protection and control of viral infections, such as SARS‐CoV‐2.^12^, ^13^

Plasma antibodies directed towards RBD generated by infection or vaccination have been widely reported to neutralise SARS‐CoV‐2 using the fragment antigen binding (Fab) portion.^2^, ^14^, ^15^ Convalescent plasma IgG and IgM have been heavily studied, with both isotypes playing a large role in the neutralising response to SARS‐COV‐2 wild‐type (WT) and VOCs.^16^, ^17^, ^18^ Limited studies suggest plasma IgA dominates the early neutralising antibody response of SARS‐CoV‐2 WT and subsequently decreases into convalescence.^19^, ^20^ Importantly, some IgA monoclonal antibodies (mAbs; monomeric and dimeric) potently neutralise SARS‐CoV‐2 WT.^21^, ^22^ However, the capacity for plasma IgA generated by infection to neutralise common RBD mutations, such as those found in VOCs, remains to be assessed.

Neutralising and non‐neutralising antibodies, including IgG and IgA, can engage with Fc gamma receptors (FcγR) and Fc alpha receptors (FcαR), respectively, on immune cells and activate Fc effector functions to clear viral infections.^23^, ^24^, ^25^ Effector functions including complement activation, phagocytosis and antibody‐dependent cellular cytotoxicity (ADCC) have been implicated in the clearance and control of SARS‐CoV‐2 infection.^12^, ^26^, ^27^ Importantly, compromised Fc effector functions and reduced neutralising potency have been linked to poorer disease outcomes in humans.^28^, ^29^ The necessity of a polyfunctional IgG antibody response has also been highlighted in animal models. While neutralisation of SARS‐CoV‐2 is essential for the protective efficacy of prophylactic IgG monoclonal antibody (mAb) therapy, additional Fc functions are required for efficacy when given as a therapeutic.^9^, ^11^ While it is well established that IgG induces Fc effector functions to SARS‐CoV‐2, the importance of plasma IgA for activation of Fc effector functions has yet to be characterised.

The functional capacity of polyclonal convalescent IgA to SARS‐CoV‐2 has yet to be investigated, especially to RBD mutations associated with VOCs. Here, we examined fractions of plasma and purified IgA and IgG, to investigate the contribution of these isotypes to the polyclonal convalescent functional antibody response to RBD and prevalent single amino acid RBD mutations.

## Results

### Robust antibody recognition and ACE2 binding inhibition by SARS‐CoV‐2 convalescent plasma

**Figure 1:**
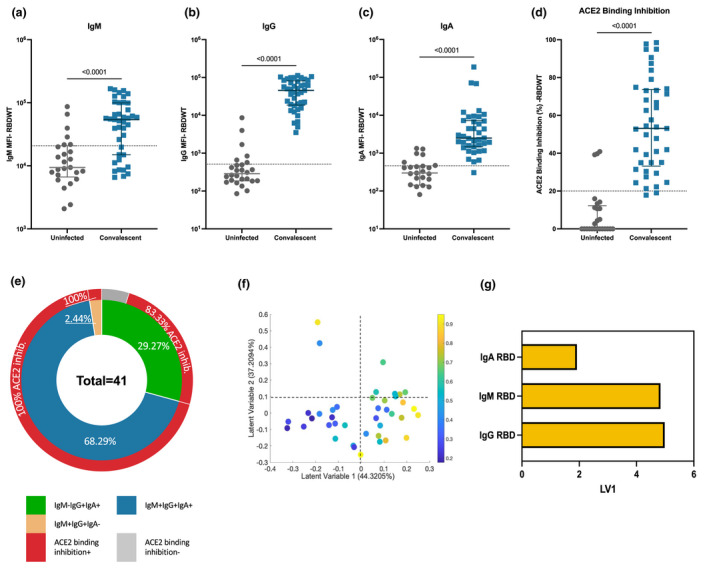
Convalescent plasma induced robust anti‐SARS‐CoV‐2 RBDWT antibody isotypes levels that inhibit ACE2 from binding RBDWT. Convalescent (*n* = 41) (blue) and uninfected control (*n* = 26) (grey) plasma (final dilution 1:100) was assessed for IgM **(a)**, IgG **(b)** and IgA **(c)** antibody binding to SARS‐CoV‐2 RBDWT via multiplex. A positive threshold (grey dotted line) was defined as the 75th percentile of antibody binding (MFI) for uninfected control plasma. Statistical analyses were determined using the Mann–Whitney *U*‐test. **(d)** RBDWT‐ACE2 binding inhibition (%) of convalescent (blue) and uninfected control (grey) plasma (diluted 1:100). A positive threshold (grey dotted line) was defined as > 20% ACE2 binding inhibition. **(e)** A pie chart outlining the percentage of subjects seropositive for anti‐RBDWT antibody isotypes [IgM^−^IgG^+^IgA^+^ (green), IgM^+^IgG^+^IgA^+^ (blue), IgM^+^IgG^+^IgA^−^ (yellow)] in the inner ring and the percentage of each seropositive subset with ACE2 binding inhibition in the outer red ring. Partial least squares regression (PLSR) were conducted to determine the multivariate relationship between anti‐RBD‐specific isotype antibodies (IgG, IgA and IgM) and % RBDWT‐ACE2 binding inhibition (the % ACE2 binding inhibition illustrated as a colour gradient legend on the right yellow—strongest to dark blue—weakest). PLSR Scores **(f)** and the loadings plot **(g).** The percentage of variance for each latent variable (LV) is shown in parentheses.

A total of 41 SARS‐CoV‐2 convalescent subjects (median age of 55; IQR: 49–61) ~ 41‐day postsymptom onset and 26 uninfected subjects (median age of 54; IQR: 24–60) donated plasma samples (Supplementary table 1). Convalescent and uninfected subject plasma were assessed for IgM, IgG and IgA binding to S1 and RBD wild‐type (RBDWT) using a SARS‐CoV‐2 multiplex bead array^30^ (Figure 1, Supplementary figure 1a–c). Most convalescent subjects generated IgM, IgG and IgA antibodies to RBDWT (IgM, 73.15% positive, median MFI = 54 086; IgG, 100% positive, median MFI = 45 745; IgA 97.56% positive, median MFI = 2495; Figure 1a–c). We also correlated age, sex and disease severity with the IgA responses (Supplementary figure 2). Although our cohort displayed a limited age range (IQR = 49–61; < 18 years, *n* = 0; > 60 years, *n* = 2), we observed a trend towards a negative correlation between RBD‐specific IgA responses and age (*r* = −0.25, *P* = 0.079; Supplementary figure 2a–d). No such associations were observed for sex or disease severity (Supplementary figure 2e and f).

To examine the ability of subject plasma to neutralise SARS‐CoV‐2, we used a multiplex RBDWT‐ACE2 binding inhibition assay that has been demonstrated to highly correlate with gold standard live microneutralisation assays.^15^ Almost all convalescent subjects (95.12%) generated significant RBDWT‐ACE2 binding inhibition (median = 73.23%; Figure 1d). ACE2 binding inhibition < 20% was considered to be below the limit of detection (Figure 1d).^15^ Significant S1‐ACE2 binding inhibition was also observed for convalescent subject plasma compared with uninfected subjects (Supplementary figure 1d and e). These data highlight that SARS‐CoV‐2 convalescent individuals generate plasma antibodies that recognise RBDWT and inhibit the binding of RBD to ACE2 as previously described.^15^

It has been well established that the titre of antibody binding to RBDWT (IgG, IgM and IgA) positively correlates with the potency of plasma neutralisation or ACE2 binding inhibition.^15^, ^17^, ^19^ Here, we separated the cohort by isotype seropositivity and RBDWT‐ACE2 binding inhibition to explore the contribution of antibody isotypes to neutralisation (Figure 1e). The majority (68.29%, 28 out of 41) of convalescent individuals were seropositive for all three isotypes (RBDWT IgM^+^IgG^+^IgA^+^) with 100% (28 out of 28) of these individuals being positive for RBDWT‐ACE2 binding inhibition (Figure 1e). 29.27% (12 out of 41) of the cohort were seronegative for IgM but were seropositive for IgG and IgA (RBDWT IgM^−^IgG^+^IgA^+^; Figure 1e). Furthermore, 83.33% (10 out of 12) of these individuals were positive for ACE2 binding inhibition (Figure 1e). A single individual (2.44% of the cohort) was seronegative for IgA but seropositive for IgM and IgG (RBDWT IgM^+^IgG^+^IgA^−^) and could mediate RBDWT‐ACE2 binding inhibition (Figure 1e). To investigate the collective contribution of anti‐RBDWT IgG, IgM and IgA to ACE2 binding inhibition, we performed partial least squares regression (PLSR; Figure 1f). Not surprisingly, plasma samples with the highest ACE2 inhibition (yellow—highest and dark blue—weakest ACE2 inhibition) predominantly clustered together across Latent Variable 1 (*x*‐axis on Figure 1f) and were strongly associated with anti‐RBDWT antibody isotype titres. Furthermore, IgG and IgM titres were the strongest correlates with ACE2 inhibition (Figure 1g). This was further confirmed by individual correlations with anti‐RBDWT antibody isotypes (Supplementary figure 3). Similar trends were observed for S1 (Supplementary figure 1a–e). Together these data suggest that while robust anti‐RBDWT IgM, IgG and IgA responses develop against SARS‐CoV‐2, these isotypes may have differential contributions to ACE2 binding inhibition.

### Reduced ACE2 binding inhibition with depletion of IgA and IgG antibodies

**Figure 2:**
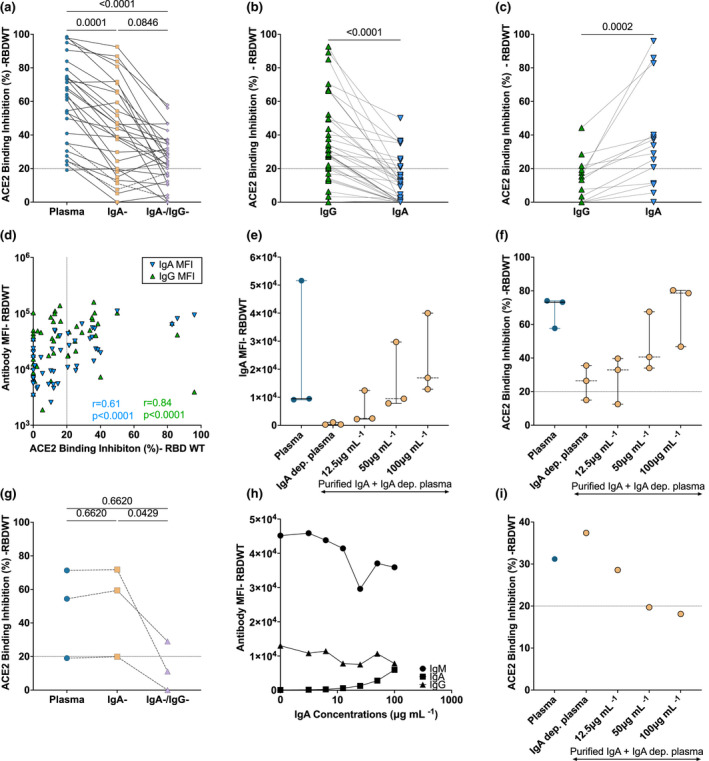
IgA from convalescent plasma induces variable levels of RBD‐ACE2 binding inhibition. RBDWT‐ACE2 binding inhibition (%) of **(a)** convalescent plasma (diluted 1:200; blue) and matched dilutions of IgA‐depleted (IgA^−^; yellow) and IgA‐ and IgG‐depleted (IgA^−^/IgG^−^; purple; *n* = 30) plasma fractions (Supplementary figure 4 describes inclusion/exclusion criteria). RBDWT‐ACE2 binding inhibition was compared for purified IgG (green) and IgA (blue) at 100 μg mL ^−1^ total purified antibody and separated as individuals where **(b)** purified IgG or **(c)** purified IgA mediated enhanced ACE2 binding inhibition (*n* = 49). **(d)** RBDWT‐ACE2 binding inhibition (%) and anti‐RBDWT IgG (green) and IgA (blue) binding (MFI) (100 μg mL ^−1^ total antibody) were correlated (nonparametric Spearman test) (*n* = 49). **(e)** RBDWT IgA binding (MFI) and **(f)** ACE2 binding inhibition (%) were assessed when increasing concentrations of purified convalescent IgA were spiked back into IgA^−^ depleted plasma (*n* = 3) (yellow, 0, 12.5, 50 and 100 μg mL ^−1^ total antibody). **(g)** A subset of samples (*n* = 3) with matched RBDWT‐ACE2 binding inhibition with IgA‐ and Ig‐A/IgG‐depletion. **(h)** IgA spiking assays were performed for the individual with the largest increase in ACE2 binding inhibition and anti‐RBDWT IgG, IgA and IgM binding (MFI) and **(i)** ACE2 binding inhibition (%) was assessed (*n* = 1). Statistical analyses of depleted plasma fractions were determined using the Friedman test with Dunn's multiple comparison test. Statistical analyses of matched purified antibody fractions were determined using the Wilcoxon signed‐rank test.

IgG plays a critical role in plasma neutralisation to various viral infections, including SARS‐CoV‐2.^15^, ^18^, ^31^ However, the contribution of plasma IgA to the neutralisation of SARS‐CoV‐2, especially to emerging RBD mutants, remains unclear.^19^, ^21^, ^32^ To define the contribution of IgG and IgA to the neutralising capacity of convalescent plasma, we depleted IgA (IgA^−^ depleted plasma) and both IgA and IgG (IgA^−^/IgG^−^ depleted plasma) from plasma to assess the capacity of antibody‐depleted plasma fractions to elicit RBDWT‐ACE2 binding inhibition (Figure 2a, Supplementary figure 4). Successful IgG and IgA depletion was confirmed by a significant reduction in IgA and IgG binding to RBDWT (*P* < 0.0001) and S1 (*P* < 0.0001) for IgA^−^ and IgA^−^/IgG^−^ depleted plasma (Supplementary figure 4f, g, i and j). 73.2% (30 out of 41) of convalescent IgA‐depleted plasma samples were successfully depleted of IgA and had < 30% loss of IgG or IgM (Supplementary figure 4b).

ACE2 binding inhibition of whole plasma (1:100 dilution) was compared with matched dilutions of IgA^−^ depleted and IgA^−^/IgG^−^ depleted plasma to investigate the contribution of IgA and IgG to convalescent plasma neutralisation (Figure 2a). IgA depletion significantly reduced the capacity for convalescent plasma to mediate RBDWT‐ACE2 binding inhibition by 22.14% (IgA depletion: median ACE2 inhibition = 41.24%, IQR: 19.19–65.41%) compared with matched whole convalescent plasma (median ACE2 inhibition = 63.38%, IQR: 35.03–75.92%, *P* = 0.0001; Figure 2a). We also investigated the contribution of IgG to convalescent plasma neutralisation. IgG depletion (IgA^−^/IgG^−^ depleted plasma) further reduced RBDWT‐ACE2 binding inhibition by 15.68% (median RBDWT‐ACE2 inhibition = 25.56%, IQR: 14.64–33.96%, *P* = 0.08) compared with IgA^−^ depleted plasma (Figure 2a). Similar trends were observed for S1 (Supplementary figure 1f and g). These data suggest both IgA and IgG contribute to the neutralising capacity of convalescent plasma for most individuals in this cohort.

### The contribution of convalescent purified IgA to RBDWT‐ACE2 binding inhibition is heterogenous

Although the above depletion studies suggest IgA contributes to convalescent plasma neutralisation for most individuals, a more definitive role of IgA or IgG in neutralisation could be revealed by purifying these fractions and measuring antibody binding using a multiplex bead array. First, we investigated the capacity for purified IgG and IgA to mediate inhibition of ACE2 binding to RBDWT at 100 μg mL ^−1^ total antibody (Figure 2b and c, Supplementary figure 5). Purified convalescent IgG (median = 27.01% IQR: 13–37–41.87%) and IgA (median = 12.17% IQR: 0–31.39) trended towards an increase in RBDWT‐ACE2 binding inhibition compared with uninfected donors (IgG median = 21.69% IQR = 14.60–29.01; IgA median = 12.57% IQR = 5.633–13.22%); however, this increase was not significant (IgA *P* = 0.7810; IgG *P* = 0.5665; Supplementary figure 5). Not surprisingly, only a smaller proportion of individuals had detectable neutralisation levels (> 20% ACE2 inhibition; purified IgA: 26.5% and purified IgG: 59.18% of individuals, Supplementary figure 4c and f–h) and then predicted by the IgA/IgG depletion experiments. This is likely due to the low concentration of purified IgA and IgG (100 μg mL^−1^) used in these assays as limited purified IgA was available.

We next compared the ACE2 inhibitory capacity of matched purified IgG and IgA for each individual at equivalent concentrations (100 μg mL^−1^). Overall, IgG mediated significantly higher ACE2 binding inhibition than IgA (*P* = 0.0045; Supplementary figure 5h). Upon further examination, 69% (34 out of 49) of individuals had lower IgA‐mediated ACE2 binding inhibition than IgG (Figure 2b, Supplementary figure 5h). Interestingly, 31% (15 out of 49) of individuals mediated stronger ACE2 binding inhibition via IgA instead of IgG (Figure 2c). Furthermore, the top three purified IgA neutralisers had comparable IgA‐mediated neutralisation when compared to the top three purified IgG neutralisers (Figure 2b and c). Similar trends were observed for S1 antibody binding and ACE2 binding inhibition for purified convalescent IgG and IgA (Supplementary figure 5a–c). We also demonstrate that the level of RBD‐specific antibody binding correlated with ACE2 binding inhibition for both purified IgA (*r* = 0.61, *P* < 0.0001) and IgG (*r* = 0.84, *P* < 0.0001; Figure 2d), suggesting that both IgG and IgA have the potential to neutralise SARS‐CoV‐2. However, this is largely dependent on anti‐RBDWT antibody titre.

To confirm the importance of antibody titre in facilitating IgA‐mediated ACE2 binding inhibition, increasing concentrations of autologous purified IgA (0, 12.5, 50 and 100 μg mL ^−1^ total antibody) were spiked back into IgA^−^ depleted plasma. This was performed for a subset of individuals with IgA‐mediated ACE2 binding inhibition (> 20%) where sufficient sample was available (*n* = 3; Figure 2e and f). IgA binding MFI (Figure 2e) and RBDWT‐ACE2 binding inhibition (Figure 2f) of IgA‐depleted plasma increased with the addition of increasing concentrations of purified IgA. A ~3‐fold increase in RBDWT‐ACE2 binding inhibition was observed with spiking of 100 μg mL^−1^ purified IgA (median ACE2 inhibition = 40.61% IQR = 34.05–67.55%) compared with IgA^−^ depleted plasma (median ACE2 inhibition = 26.43% IQR = 14.98–35.50%; Figure 2f). These results highlight that while IgA does contribute to convalescent plasma neutralisation, the capacity to neutralise is highly dependent on IgA antibody titre.

Intriguingly, we identified a small subset of individuals (*n* = 3), where depletion of IgA resulted in a small non‐significant increase in ACE2 inhibition (Figure 2g; plasma: median ACE2 inhibition = 54.41%, IgA^−^ depleted plasma: median ACE2 inhibition = 59.41%). Plasma IgA has previously been described to block IgG‐mediated functions, including neutralisation.^33^, ^34^, ^35^, ^36^ We performed IgA spiking assays to investigate whether plasma IgA could block the binding of other antibody isotypes to RBDWT. The individual with the strongest RBDWT‐ACE2 binding inhibition (CP30) was selected, and purified IgA was spiked at increasing concentrations (0, 12.5, 50 and 100 μg mL^−1^ total antibody) back into IgA^−^ depleted plasma. Addition of purified IgA into IgA^−^ depleted plasma for this individual decreased IgM binding to RBDWT (Figure 2h) and decreased ACE2 binding inhibition (Figure 2i) in a titre‐dependent manner. Purified IgA at the highest concentration tested (100 μg mL ^−1^) decreased IgM binding by 0.2‐fold (Figure 2h) and decreased RBDWT‐ACE2 binding inhibition by 0.5‐fold compared with IgA^−^ depleted plasma (Figure 2i). Our results suggest that in rare individuals IgA may block RBD‐specific IgM binding, thus reducing ACE2 binding inhibition. Clearly, due to the very small sample size, this phenomenon needs to be explored in greater detail in a much larger cohort before definitive conclusions can be made.

### Antibody binding profiles of SARS‐CoV‐2 convalescent purified IgG and IgA to RBD single mutants

**Figure 3:**
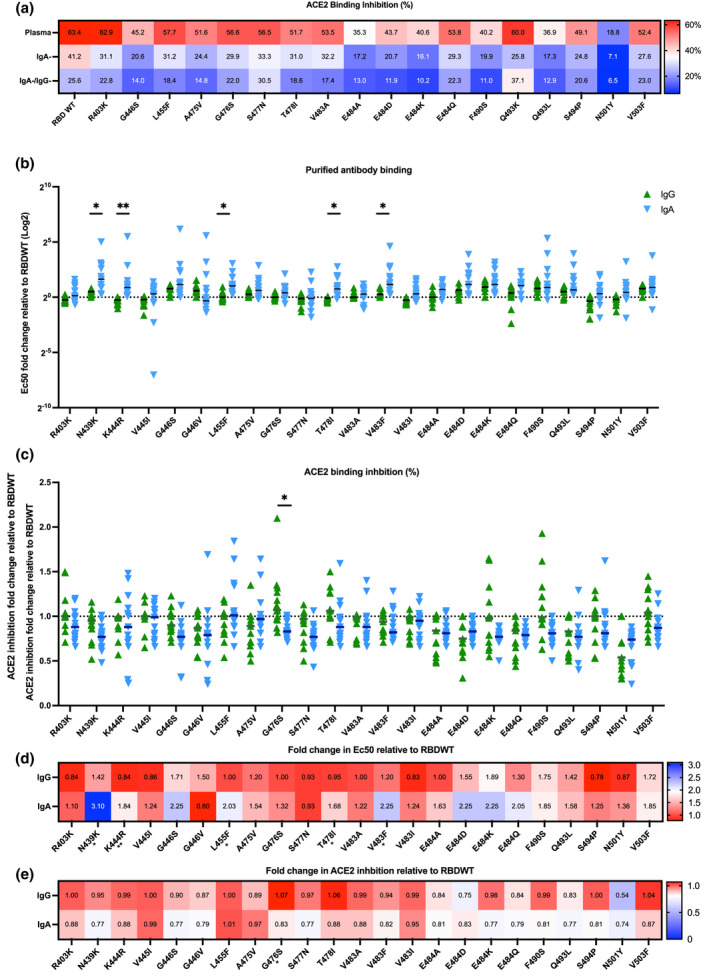
IgA and IgG have comparable antibody binding and ACE2 binding inhibition to prevalent RBD single mutants. **(a)** A heatmap of the mean ACE2 binding inhibition (%) for RBDWT and 18 RBD single mutants of matched complete plasma, IgA^−^ depleted and IgA^−^/IgG^−^ depleted plasma (*n* = 30). **(b)** The fold change in EC_50_ for purified IgA (blue) and IgG (green) antibody binding relative to RBDWT for 23 different RBD single mutants (*n* = 13) **(c)** The fold change in RBD‐ACE2 binding inhibition for purified IgA and IgG relative to RBDWT for 23 different RBD single mutants (*n* = 13). Statistical analyses were performed with the paired *t*‐test adjusting for multiple comparisons using the Holm‐Šídák method (* = *P* < 0.05, ** = *P* < 0.01). The median fold changes in **(d)** antibody EC_50_ binding and **(e)** RBD‐ACE2 binding inhibition relative to RBDWT for each RBD mutant are summarised as heatmaps.

SARS‐CoV‐2 variants are rapidly emerging, which possess a constellation of amino acid mutations within the RBD, some of which confer increased ACE2 affinity and antibody escape.^37^, ^38^, ^39^ The capacity for IgG mAbs, convalescent plasma and plasma IgG to recognise different RBD mutations has been widely characterised.^15^, ^18^ However, the capacity of convalescent purified IgA to recognise different RBD mutations is yet to be reported. To explore the impact of RBD mutations on IgA‐ and IgG‐driven ACE2 binding inhibition, plasma, IgA^−^ depleted plasma and IgA^−^/IgG^−^ depleted plasma were assessed for ACE2 binding inhibition to a panel of 23 single amino acid RBD mutants, using a previously published competitive RBD‐ACE2 inhibition multiplex assay.^13^ As expected, reduced plasma ACE2 binding inhibition was observed for a number of RBD single amino acid mutations including E484K (present in Beta and Gamma VOC) and N501Y (present in Beta, Gamma and Omicron) when compared to RBDWT.^40^ Furthermore, depletion of IgA and IgG reduced RBD‐ACE2 binding inhibition across all RBD variants, suggesting that IgA and IgG isotypes contribute to the recognition of RBD variants (Figure 3a, Supplementary figure 6a).

To further characterise the IgA‐ and IgG‐driven ACE2 binding inhibition to RBD mutations, individuals with IgA‐mediated RBDWT‐ACE2 binding inhibition (median: 28.31%, *n* = 13) were characterised further. Matched purified IgG and IgA were assessed for antibody binding and ACE2 binding inhibition to 23 common RBD single amino acid mutations (Figure 3b–e). First, antibody binding relative to RBDWT for 23 RBD single mutants was calculated using EC_50_ values obtained from the normalised antibody binding MFI for matched purified IgA and IgG samples (titrations up to 100 μg mL^−1^; Figure 3b and d, Supplementary figure 6b). Importantly, for 18 of the 23 mutants (78.26%) there was no significant difference in antibody binding for purified IgG and IgA (Figure 3b and d). Overall, IgG trended towards better antibody binding (lower EC_50_) than IgA, with 5 RBD mutants (L455F (*P* = 0.0153), N439K (*P* = 0.0153), T478I (*P* = 0.0107), K444R (*P* = 0.0056) and V483F (*P* = 0.032), all *P*‐values adjusted for multiple comparison) having significantly greater IgG recognition than IgA recognition (Figure 3b and d). Interestingly, the RBD mutant G446V showed a trend towards (adjusted *P* = 0.0956) better IgA recognition relative to RBDWT (median EC_50_ = 157.7 IQR = 68.86–180.9) than that to IgG (median EC_50_ = 246.5 IQR = 108.9–400; Figure 3b and d, Supplementary figure 6b). Notably, we observed a larger range in fold change for IgA binding (median range of fold change across all variants = 7.017) relative to RBDWT for the 23 RBD mutants than for IgG binding (median range of fold change across all variants = 0.64), further highlighting the heterogeneity in IgA responses (Figure 3b). Importantly, these data suggest IgG and IgA have similar capacities to recognise RBD mutations.

We next investigated whether differences in antibody recognition by IgG and IgA translated to a change in ACE2 binding inhibition. We assessed ACE2 binding inhibition of purified IgA and IgG for the same subset of individuals (*n* = 13) to 23 RBD single mutants at a single concentration (100 μg mL ^−1^ total antibody; Figure 3c and e). Purified IgG had significantly increased ACE2 binding inhibition relative to RBDWT compared with purified IgA for 1 RBD mutation (G476S) (*P* = 0.011; Figure 3c). Increased ACE2 binding inhibition of G476S by purified IgG may be due to enhanced recognition of G476S compared with purified IgA (not significant; Figure 3b, Supplementary figure 6c). This suggests that IgG and IgA may recognise different epitopes of G476S RBD. Importantly, for 22 of the 23 mutants (95.65%) there were marginal differences in ACE2 binding inhibition for purified IgG and IgA, suggesting that IgG and IgA can neutralise most RBD mutants similarly. Taken together, our data suggest that purified convalescent IgA and IgG have similar capacities to recognise most RBD mutations assayed here. Intriguingly, where significant differences were observed in IgG and IgA antibody recognition, ACE2 binding inhibition or neutralisation was marginally impacted.

### SARS‐CoV‐2‐specific IgG mediates Fc effector functions of convalescent plasma

**Figure 4:**
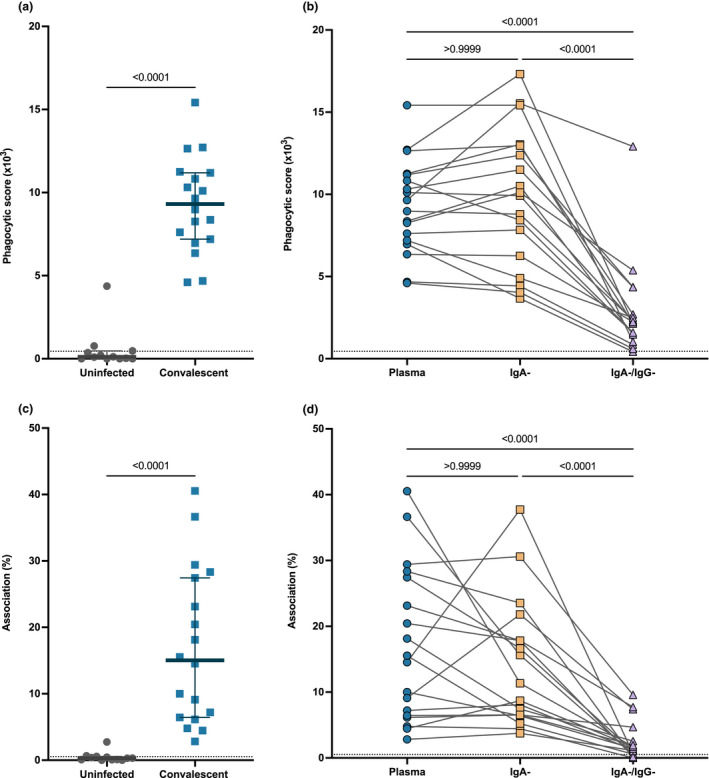
Depletion of IgG substantially reduced the Fc functional capacity of convalescent plasma. **(a)** Antibody‐dependent phagocytosis (ADP) of S‐conjugated beads for a subset of convalescent individuals (blue; *n* = 18) and uninfected control plasma (grey) (*n* = 12; diluted 1:900). **(b)** Comparison of ADP of S‐conjugated beads from matched convalescent subject plasma (blue), IgA‐depleted plasma (IgA^−^; yellow) and IgA‐ and IgG‐depleted plasma (IgA^−^/IgG^−^; purple) (*n* = 18). **(c)** Fc gamma receptor (FcyR)‐dependent association of THP‐1 cells with Ramos S‐orange cells mediated by convalescent plasma (blue; *n* = 18) and uninfected control plasma (grey) (*n* = 12; diluted 1:100). **(d)** FcyR‐dependent association of THP‐1 cells with Ramos S‐orange cells from matched convalescent subject plasma (blue), IgA‐depleted plasma (IgA^−^; yellow) and IgA‐ and IgG‐depleted plasma (IgA^−^/IgG‐; purple) (*n* = 18). The grey dotted lines denote the negative threshold for cell association (< 2%) and ADP (3 x 10^3^) calculated as the mean response of negative subject plasma plus 2 standard deviations. Statistical analyses were performed with the Kruskal–Wallis test followed by the Dunn's multiple comparisons test.

Antibody Fc portions can engage with FcyR on monocytes to mediate antibody‐dependent phagocytosis (ADP). Anti‐SARS‐CoV‐2 IgG mAbs and plasma IgG has been reported to induce robust ADCP responses; however, the Fc functional role of plasma IgA in SARS‐CoV‐2 remains unknown. Here, we investigated the capacity of convalescent plasma to induce ADP using a previously described SARS‐CoV‐2 ADP bead‐based assay.^27^ We measured the antibody‐mediated uptake of spike trimer (S)‐conjugated fluorescent beads by THP‐1 monocytes with a subset of convalescent (*n* = 18) and uninfected control plasma (*n* = 12; Figure 4a). All convalescent subjects had higher ADP (median phagocytic score = 9.31 × 10^3^ IQR = 7.14 × 10^3^–11.20 × 10^3^) than that for uninfected subjects (median phagocytic score = 0.11 × 10^3^ IQR = 0 × 10^3^–0.45 × 10^3^, *P* < 0.0001; Figure 4a). To determine the contribution of IgA and IgG to the ADP response against SARS‐CoV‐2, we measured ADP activity mediated by IgA^−^ depleted and IgA^−^/IgG^−^ depleted plasma. (Figure 4b). Depletion of IgA had no effect on the ADP capacity of the convalescent plasma (convalescent plasma, median phagocytic score = 9.31 × 10^3^; IgA‐depleted plasma, median phagocytic score = 10.01 × 10^3^, IQR = 5.91 × 10^3^–12.98 x 10^3^, *P* > 0.999) (Figure 4b). Conversely, subsequent depletion of IgG (IgA^−^/IgG^−^ depleted plasma) (median = 2.20 x 10^3^, IQR = 1.36 × 10^3^–3.11 × 10^3^) resulted in a significant loss of 76.37% ADP activity compared with convalescent plasma (*P* < 0.0001; Figure 4b). These data highlight the importance of the IgG antibody isotype in the Fc functional capacity of SARS‐CoV‐2 convalescent plasma.

In addition to the uptake of antibody opsonised virions, antibodies can also mediate cell association and trogocytosis of infected cells expressing SARS‐CoV‐2 spike. Using an established cell association assay,^27^ we measured antibody‐mediated association of THP‐1 monocytes with cells expressing SARS‐CoV‐2 spike (Ramos S‐orange cells) following incubation with convalescent (*n* = 18) and uninfected subject plasma (*n* = 12; Figure 4c). Antibody‐mediated association was detected in all convalescent subjects (median association = 15.04% association IQR = 6.63–27.66%) when compared to uninfected subjects (median association = 0.3% association IQR = 0.08–0.53%, *P* < 0.0001; Figure 4c). We then investigated the contribution of IgA and IgG to cell association/trogocytosis through depletion of IgA (IgA^−^ depleted plasma) and IgG (IgA^−^/IgG^−^ depleted plasma) from convalescent plasma (Figure 4d). Depletion of IgA (median association = 10.02%; IQR = 6.50–18.83%) did not significantly alter the cell association mediated by convalescent plasma (median association = 15.04% association IQR = 6.63–27.66%, *P* > 0.99; Figure 4d). However, depletion of IgG (IgA/IgG‐depleted plasma) (median association = 1.33% association IQR = 0.61–3.10%) resulted in a significant loss of 90.53% cell association relative to convalescent plasma (median association = 15.04% association IQR = 6.63–27.66%, *P* < 0.0001; Figure 4d). These data further support the role of IgG convalescent plasma for an effective Fc functional response to SARS‐CoV‐2 virions and virus‐infected cells.

## Discussion

Neutralising antibodies are a strong correlate of protection for most viral vaccines, including SARS‐CoV‐2 vaccines.^40^, ^41^ IgM and IgG have been widely implicated in the effective neutralisation of SARS‐CoV‐2.^2^, ^15^, ^17^, ^42^ However, the study of anti‐SARS‐CoV‐2 IgA responses have been relatively neglected throughout the pandemic.^32^ The plasma IgA response to SARS‐CoV‐2 is relatively transient in nature peaking during acute infection and dominates the acute plasma neutralising response (half‐life of 42 days in the first 60 days and > 1000 days from 60–160‐day postsymptom onset).^2^, ^19^ Using our surrogate neutralisation assay, we show that anti‐RBD plasma IgA contributes to the neutralising capacity during early convalescence for many individuals, in addition to IgG and IgM isotypes. We also found decreased neutralisation when IgA was depleted from convalescent plasma supporting observations made by Gasser *et al*.^17^ and reinforcing IgA's contribution to SARS‐CoV‐2 neutralisation. Furthermore, for the first time, we show that IgA depletion and IgG depletion also reduces neutralisation for several prevalent RBD mutations.

Intriguingly, we observed IgA‐associated and IgA‐independent neutralising antibody responses in convalescent plasma. An IgA‐independent neutralising response was observed in 30.3% of individuals with no change in ACE2 binding inhibition when IgA was depleted. While 69.7% of individuals showed a significant reduction in ACE2 binding inhibition when IgA was depleted, suggesting IgA‐associated neutralisation. Furthermore, like Wang *et al*.^21^ we observed a subset of convalescent individuals who mediated superior neutralisation with IgA when compared to IgG. Although, purified IgG mediated higher RBD‐ACE2 inhibition than purified IgA for most convalescent donors. While we show that IgG and IgA contribute to convalescent plasma neutralisation, depletion of IgG and IgA did not abolish this response in most individuals, further reinforcing the importance of IgM in the convalescent plasma neutralising response to SARS‐CoV‐2.^17^, ^42^ Our data further support the finding that IgA is capable of neutralising SARS‐CoV‐2 WT. However, this response is highly heterogenous and is likely to be due to variability in IgA titres generated against RBD.

The potency of the IgA neutralising response was confirmed to be titre dependent using IgA spiking assays. However, large heterogeneity was observed in the magnitude of the SARS‐CoV‐2 IgA response amongst individuals. Given that IgA is broadly cross‐reactive towards human coronaviruses in milk, saliva and plasma,^32^, ^43^, ^44^, ^45^ pre‐existing IgA^+^ memory B cells for human coronaviruses may recognise SARS‐CoV‐2 RBD with higher affinity and undergo somatic hypermutation to create a potent and robust IgA response in some individuals.^46^, ^47^ Furthermore, cytokine environment and/or host genetics (e.g. age or gender) are known to influence preferential class switching to IgG or IgA.^48^ This cohort was not powered to correlate IgA with age or severity reliably (Supplementary figure 2) and cytokine analysis or sequencing of gamma and alpha genes were not in the scope of this project. However, others have observed higher anti‐SARS‐CoV‐2 IgA in patients presenting with severe disease (similar to IgG) and in elderly individuals.^30^, ^49^, ^50^ Additionally, the cytokine profiles induced by SARS‐CoV‐2 vary with disease severity^51^ and are likely to impact class switching.^48^, ^52^ Therefore, human coronavirus infection history, cytokine environment and host genetics are likely to influence the magnitude of the IgA response to SARS‐CoV‐2.

Intriguingly, we also observed decreased neutralisation and IgM binding to RBDWT for a single individual when increasing concentrations of purified IgA were spiked back into IgA‐depleted plasma. Although this is a single case study and cannot be used to draw any conclusions, these data suggest IgA has the potential to block RBD‐specific IgM antibodies from binding and neutralising SARS‐CoV‐2 in extremely rare cases. IgA binding to non‐neutralising epitope(s) may block the binding of potent neutralising IgM antibodies. Similarly, IgA has been shown to block IgG binding to Epstein—Barr virus (EBV) and reduce ADCC,^36^ block IgG complement‐mediated lysis of group C *Neisseria meningitidis* ^34^ and block IgG and IgM complement activation through independent mechanisms.^35^ Tomaras *et al*.^33^ also observed epitope competition of an anti‐HIV IgA monoclonal antibody for protective IgG derived from HIV‐1 vaccinated individuals, resulting in reduced functional activity. However, the IgA blocking phenomenon observed here warrants further investigation in a much larger cohort.^33^ Importantly, the vast majority of individuals have the capacity to neutralise wild‐type SARS‐CoV‐2 when a robust IgA response is generated.

Constellations of single amino acid substitutions within the RBD are found in the variants of concern, which have displaced the ancestral SARS‐CoV‐2 strain. Single amino acid substitutions can alter the stability and potentially the structure of the RBD, thus may reveal or hide epitopes from different isotypes and/or subclasses of antibodies.^37^, ^53^ It is important to understand antibody isotype (IgG and IgA) responses to these single‐site mutations to aid in next‐generation vaccine design. For individuals with IgA‐skewed neutralisation, we compared the capacity for polyclonal purified convalescent IgA to recognise and neutralise single amino acid RBD mutants compared with IgG. We observed minor differences in purified antibody isotype binding to various RBD single mutants, despite the small sample sizes and the polyclonal nature of these convalescent responses. A trend towards preferential IgG binding to many mutants was observed, with significantly increased IgG binding to five mutations. Although IgG recognised 5 of the 23 RBD mutations significantly better than IgA, this did not translate to differences in ACE2 binding inhibition. This may provide useful insights into the amino acid epitopes in RBD, which have a minor influence upon ACE2 binding/inhibition. However, it should be noted that most of the differences observed in isotype binding were small (median fold change relative to RBDWT IgG = 1.13, IgA = 2.36) and therefore may explain the lack of change in ACE2 binding inhibition for many RBD mutations. Furthermore, the Fc portion of the antibody can affect the fine epitope specificity (binding affinity/epitope recognition) of the Fab region leading to slight variations in IgG and IgA recognition of RBD mutants.^22^, ^54^, ^55^, ^56^ However, our results suggest that antibody isotype (IgG or IgA) contributes minimally to the level of recognition and neutralisation of emerging RBD variants. Other factors such as antibody affinity or titre are more important.^22^, ^23^ Critically, our results show that convalescent IgG and IgA broadly recognise similar RBD epitopes with minimal differences in the ability to neutralise RBD mutants.

In addition to antibody neutralisation, Fc effector functions such as phagocytosis are important for control and clearance of SARS‐CoV‐2 infection.^9^, ^11^, ^57^, ^58^ We show that convalescent plasma can mediate ADP, supporting observations made by others.^58^ Interestingly, IgA depletion from convalescent plasma did not impact Fc effector functions by THP‐1 monocytes. Conversely, Butler *et al*. (2021)^58^ used regression analysis to suggest convalescent IgA contributes to the Fc functional capacity of convalescent plasma. In this study, we used THP‐1 cells, which express relatively low amounts of FcαR, and thus, we may not capture the functional potential of IgA. Importantly, depletion of IgG abolished Fc effector functions in our assays, further reinforcing the importance of IgG in the polyfunctional (neutralising and Fc effector function) antibody response of convalescent plasma.

While this study focussed on circulating IgA, secretory IgA (sIgA) plays an important role in viral immunity at mucosal surfaces through immune exclusion and viral clearance.^59^, ^60^ sIgA can be found in mucosal secretions including saliva, tears and bronchoalveolar lavage (BAL).^21^, ^61^ sIgA is dimeric in nature and has enhanced neutralisation of SARS‐CoV‐2 compared with monomeric IgA.^21^, ^61^ While sIgA is predominantly secreted locally at mucosal surfaces, the remaining IgA is transcytosed from circulation into the mucosal lumen.^62^ Anti‐SARS‐CoV‐2 IgA has been detected in the BAL, saliva and tears of humans^19^, ^62^, ^63^, ^64^, ^65^, ^66^ with BAL and salivary antibodies able to neutralise SARS‐CoV‐2 following infection.^19^ Recently, mucosal IgA but not IgG was suggested to be a correlate of protection for SARS‐CoV‐2 breakthrough infection following two doses of an mRNA SARS‐CoV‐2 vaccine, even with poor induction of an IgA response in most individuals.^67^ Furthermore, animal studies have highlighted the importance of mucosal IgA in protection from SARS‐CoV‐2 following mucosal or heterogenous vaccination.^64^, ^68^, ^69^, ^70^, ^71^ Intriguingly, a recent preprint has observed elevated dimeric IgA specific for RBD during acute infection.^72^ Furthermore, the persistence of a viral reservoir in the gut of individuals with long covid^73^ suggests that mucosal IgA, including dimeric forms, may be passing into the plasma during acute infection. Future studies could potentially examine plasma as a biomarker of acute infection and long COVID. Importantly, Sterlin *et al*.^19^ detected monomeric IgA in BAL following acute SARS‐CoV‐2 infection in humans, suggesting circulating IgA is transcytosed into the lungs where IgA may exert effector functions. Therefore, it is reasonable to suggest that plasma IgA can provide a solid indicator of the mucosal IgA response to SARS‐CoV‐2.

Overall, we find that convalescent plasma IgA can recognise and block ACE2 engagement with RBDWT and an array of RBD mutants in a comparable manner to IgG when robust IgA titres are induced. Furthermore, the convalescent IgA neutralising response is highly heterogenous between individuals, with a third of the cohort inducing stronger IgA‐mediated ACE2 binding inhibition than that of IgG at equivalent concentrations. Dissecting the IgA response in the context of vaccination and to variants of concern is essential to further understand the importance of IgA in a protective polyclonal antibody response.

## Methods

### Ethics statement

The study protocols were approved by the University of Melbourne Human Research Ethics Committee (#2056689), and all associated procedures were carried out in accordance with the approved guidelines. All participants provided written informed consent in accordance with the Declaration of Helsinki.

### Human subjects

Participants who had recovered from COVD‐19 during the first wave of the pandemic in Melbourne, Australia, March–May 2020, were recruited as previously described.^2^ Convalescent subjects were confirmed to have had COVID‐19 by returning a positive PCR test during early infection or tested positive for SARS‐CoV‐2 serology (both Spike trimer and RBD) confirming prior exposure as previously reported.^74^ Uninfected controls with no COVID‐19 symptoms were also recruited during the first wave of COVID‐19 and were confirmed as seronegative. Whole blood was collected with sodium heparin. The plasma fraction was then collected and stored at −80°C. Cohort characteristics for convalescent and healthy controls are outlined in Supplementary table 1.

### SARS‐CoV‐2 bead‐based multiplex assay

The SARS‐CoV‐2‐specific antibody isotypes (IgM, IgG and IgA1) were assessed using a multiplex assay as previously described.^30^ Briefly, the bioplex magnetic carboxylated bead (Bio‐Rad, Gladesville, Australia) mixture containing 700 beads per bead region and diluted plasma or purified antibody was added to each well in a black clear bottom nonbinding 384‐well microplate (Greiner Bio‐One, Kremsmünster, Austria). Phycoerythrin (PE)‐conjugated mouse anti‐human pan‐IgG and IgA1 (Southern Biotech, Birmingham, USA; 1.3 μg mL^−1^) were added to detect SARS‐CoV‐2‐specific antibodies. For IgM detection, biotinylated mouse anti‐human IgM (mAb MT22; MabTech, Cincinnati, USA) was added at 1.3 μg mL^−1^. Following incubation, streptavidin R‐Phycoerythrin conjugate (SAPE) (Thermo Fisher Scientific, Invitrogen, Scoresby, Australia) at 1 μg mL^−1^ was added. The plate was read via the FlexMap 3D (Luminex, Austin, Texas), and binding of PE‐detectors was measured to calculate the median fluorescence intensity (MFI). Background was corrected for by subtracting the MFI of BSA‐blocked beads for each well. Titrations of pooled convalescent plasma and an anti‐SARS‐CoV‐2 RBD neutralising human IgG1 antibody (SAD‐S35, ACRO Biosystems, Newark, USA) were included as positive controls, and uninfected subject plasma was included as negative controls. A single dilution was used for plasma and antibody‐depleted plasma. EC_50_s were calculated for purified IgG and IgA where appropriate using normalised antibody binding.

### RBD‐ACE2 binding inhibition multiplex bead‐based assay

The RBD‐ACE2 binding inhibition assay was performed as previously described.^15^ Briefly, an array of SARS‐CoV‐2 antigens including S1 (Sino Biological, Beijing, China), RBD wild‐type (WT) (B; wild‐type, Wuhan) and 18 RBD single mutants (kindly provided by Wai‐Hong Tham, WEHI, Melbourne, Australia) were used in Figure 3a. Five additional RBD mutants were included in Figure 3b–e. The bead mixture containing 700 beads per bead region was added to each well (20 μL) with biotinylated Avitag‐ACE2 (kindly provided by Dale Godfrey, Nicholas Gherardin and Samuel Redmond, The Peter Doherty Institute for Infection and Immunity, Melbourne, Australia) at a final concentration of 12.5 μg mL^−1^ per well in a 384‐plate. Dilutions of plasma or purified antibodies were incubated with beads and Avitag‐ACE2 and then washed. Biotinylated Avitag‐ACE2 was detected using SAPE at 4 μg mL^−1^ followed by PE‐Biotin amplifier (Thermo Fisher Scientific) at 10 μg mL^−1^. Plates were washed and acquired on a FlexMap 3D (Luminex). Anti‐SARS‐CoV‐2 RBD neutralising human IgG1 antibody (SAD‐S35, ACRO Biosystems) was included as a positive control, in addition to COVID‐19 negative plasma and buffer‐only negative controls. The MFI of bound ACE2 was measured after background subtraction of no ACE2 controls. Maximal ACE2 binding MFI was determined by ACE2 only controls. The % ACE2 binding inhibition was calculated as 100% − (% ACE2 binding MFI per sample/Maximal ACE2 binding).

### Human IgG and IgA ELISA

Purified IgG and IgA concentrations were quantified using human anti‐IgG kit (cat #3850‐1 AD‐6 Mabtech) or anti‐IgA kit (cat #3860‐1 AD‐6 Mabtech), respectively, as per the manufacturer's instructions. Briefly, anti‐IgG or IgA capture antibody was coated on Maxisorb 96‐well plates (Nunc) overnight at 4°C. Plates were washed with PBS containing 0.05% Tween20 (PBST) and blocked with 1% BSA/PBST for 2 h. The plates were washed, and purified IgG or IgA antibodies were titrated twofold from 1:20 000 for a minimum of 4 points. To check for IgG contamination, purified IgA antibodies were tested at 1:1000 and 1:2000 dilutions. Similarly, purified IgG antibodies were tested at 1:1000 and 1:2000 to check for IgA contamination. Antibodies and respective standards were incubated for 2 h at RT before being washed. The secondary‐ALP conjugated antibody was added to each well and incubated at RT for 1 h. The plate was washed, and the substrate p‐nitrophenyl‐phosphate (pNPP) was added and left to develop (~ 30 min). The optical density at 405 nm was read using Thermo Fisher Multiskan Ascent plate reader. Purified IgA and IgG were confirmed to be free of contamination from the other isotype if the OD was less than the no antibody control OD plus 2 standard deviations.

### Antibody purification and depletion

IgA purification and depletion from plasma samples were performed via affinity chromatography using peptide M agarose (Jomar Life Research, Scoresby, Australia) following the manufacturer's instructions (Supplementary figure 4a and b). Briefly, peptide M agarose was added to 1‐mL filter columns (Thermo Fisher Scientific) and washed three times with PBS. A volume of 300 μL of plasma was incubated with peptide M columns for 45 min on an orbital at room temperature. Depleted IgA plasma fractions were collected by spinning at 1000 *g* for 1 min after incubation. Purified IgA was eluted with low pH (pH 2.8) by IgG Elution Buffer (Thermo Fisher Scientific). Elutions were neutralised using Tris M pH 8.0 (Thermo Fisher Scientific).

IgG was purified from 100 μL of IgA^−^ depleted plasma using 96‐well Protein G HP MultiTrap (GE Healthcare, Chicago, USA) following the manufacturer's instructions (Supplementary figure 4a and b). Purified IgA was also passed through the MultiTrap to remove any IgG contamination. Briefly, IgA^−^ depleted plasma and purified IgA were diluted 1:1 in antibody binding buffer and added to the MultiTrap. Samples were incubated for 30 min at RT while shaking. The plate was centrifuged, IgA and IgG (IgA^−^/IgG^−^) depleted plasma and purified IgA was collected. The plate was washed with antibody binding buffer before elution of IgG with 200 μL of elution buffer (GE Healthcare). Purified IgG was collected via centrifugation at 200 *g* for 2 min and neutralised to pH 7 using neutralisation buffer (GE Healthcare). Elution was performed three times, and purified IgG, IgA^−^ depleted and IgA^−^/^−^ depleted plasma was buffer exchanged into PBS and concentrated to original starting volume.

### Antibody depletion quality control and dilution matching

#### Matching dilutions to account for the loss of antibody during depletion process

Anti‐RBDWT IgG (Supplementary figure 3c) and IgM (Supplementary figure 3d) binding was assessed for plasma, IgA^−^ depleted and IgA^−^/IgG^−^ depleted plasma via multiplex. During the depletion process, a median loss of 48.88% of IgG (Supplementary figure 3c, median MFI = 21 150, *P* = 0.0160) and 36.95% of IgM (Supplementary figure 3d, median MFI = 26 544, *P* = 0.0002) was observed following the depletion of IgA (IgA^−^ depleted plasma) and IgG (IgA^−^/IgG^−^ depleted plasma), respectively, compared with whole plasma (median IgG MFI = 41 377, median IgM MFI = 42 101; Supplementary figure 3c and d). To ensure fair comparisons between plasma and depleted plasma fractions, plasma and depleted plasma samples were titrated and anti‐RBDWT IgG and IgM binding MFI was determined via multiplex (Supplementary figure 3e). IgA^−^ depleted and IgA^−^/IgG^−^ depleted plasma dilutions were chosen by matching IgG or IgM MFI, respectively, to the IgG or IgM binding MFI of whole plasma at a dilution of 1:100. Final dilutions are outlined in Supplementary table 2. These dilutions were used for all multiplex assays. Depleted plasma samples with > 30% loss in IgG or IgM following the matching of dilutions were excluded from this study (Supplementary figure 3b).

#### Quality control testing of antibody‐depleted plasma

Successful depletion IgG and IgA was confirmed for matched dilutions via IgG SARS‐CoV‐2 RBDWT multiplex (Supplementary figure 3f–k, Supplementary table 2). This method detected the remaining antigen‐specific IgG or IgA with high sensitivity allowing a stringent threshold for exclusion of samples with un‐successful antibody depletion. Anti‐RBDWT IgG or IgA binding MFI of depleted plasma was compared with whole plasma for each subject, and the percentage reduction in IgG and IgA binding was calculated. Sufficient depletion was defined as > 70% depletion of IgG or IgA (i.e. > 70% reduction in anti‐RBDWT IgG MFI compared with plasma IgG MFI; Supplementary figure 3f–k). Plasma, IgA^−^ depleted and IgA^−^/IgG^−^ depleted plasma samples with < 70% depletion of IgA or IgG from whole plasma were all excluded from this study (Supplementary figure 3b). Only subjects with all three plasma fractions (plasma, IgA^−^ depleted and IgA^−^/IgG^−^ depleted plasma) were included in final analysis (*n* = 30; Supplementary figure 3b).

### IgA spiking assay

We performed IgA spiking assays as proof of concept that IgA contributes to ACE2 binding inhibition in a dose‐dependent manner. Using the RBDWT‐ACE2 binding inhibition assay and the SARS‐CoV‐2 multiplex assay, up to 100 μg mL ^−1^ (0, 12.5, 50 and 100 μg mL^−1^) of autologous purified IgA was spiked back into IgA^−^ depleted plasma for three individuals. The impact of purified IgA on ACE2 binding inhibition and antibody binding for IgA‐depleted plasma was observed.

### Fc effector functional assays

#### Cell culturing

THP‐1 monocytes (ATCC, Manassas, USA) and Ramos cells expressing mOranage2 SARS‐CoV‐2 spike trimer (Ramos S‐orange cells) (kindly provided by Wen Shi Lee, The Peter Doherty Institute for Infection and Immunity, Melbourne, Australia) were cultured in RPMI 1640 with 10% FCS (RF10) under recommended cell culture conditions (37°C with 5% CO_2_).^27^ THP‐1 monocytes and Ramos S‐orange cells were maintained below a cell density of 0.3 × 10^4^ and 1.0 × 10^4^, respectively. Flow cytometry was used to confirm stable expression of FcγRI (CD64), FcγRII (CD32) and FcαR (CD89) on THP‐1 monocytes and stable expression of SARS‐CoV‐2 spike trimer (S‐trimer) and mOrange2 for Ramos S‐orange cells. Cell viability was determined using trypan blue exclusion and morphology confirmed using light microscopy prior to assay preparation.

#### Antibody‐dependent bead‐based phagocytosis assay

An antibody‐dependent bead‐based phagocytosis assay was used as previously described.^27^ Briefly, SARS‐CoV‐2 spike trimer protein (S‐trimer) (kindly provided by Adam Wheatly, The Peter Doherty Institute for Infection and Immunity, Melbourne, Australia) was biotinylated and coupled to 1‐μm fluorescent NeutrAvidin Fluospheres (beads; Invitrogen, Waltham, USA) overnight at 4°C. S‐trimer‐coated beads were washed and diluted 1:100 in 2% BSA/PBS. 10 μL of diluted S‐trimer‐coated beads were incubated with plasma diluted 1:100 for 2 h at 37°C in a 96‐well U‐bottom cell culture plate. THP‐1 monocytes (1 x 10^4^) were added to opsonised beads and incubated for 16 h under cell culture conditions. THP‐1 monocytes were fixed, and cells were acquired by flow cytometry on a BD LSR Fortessa with a high‐throughput sampler attachment (HTS) (refer to Supplementary figure 7a for the gating strategy). The data were analysed using FlowJo 10.7.1, and a phagocytosis score ([% bead positive cells×mean fluorescent intensity]/10^3^) was calculated as previously described.^75^

#### THP‐1 and Ramos S‐orange cell association assay

A THP‐1 and Ramos S‐orange cell association was used as previously described.^27^ Briefly, THP‐1 monocytes were stained with CellTrace™ Violet (CTV; Life Technologies, Carlsbad, USA) as per the manufacturer's instructions. Concurrently, Ramos S‐orange cells (1 x 10^4^) were added to each well in a 96‐well V‐bottom cell culture plate. Plasma was diluted to 1:900 with the Ramos S‐orange cells and incubated for 30 min under cell culture conditions. Opsonised Ramos S‐orange cells were washed by centrifugation, and CTV‐stained THP‐1 monocytes (1 x 10^4^) were added for a final 1:1 ratio of THP‐1 monocytes to Ramos S‐orange cells. Opsonised Ramos S‐orange cells and THP‐1 monocytes were incubated for 1 h under cell culture conditions before fixation. Cells were acquired by flow cytometry using the BD LSR Fortessa with a HTS, and the data were analysed using FlowJo 10.7.1 (refer to Supplementary figure 7b for the gating strategy). The percentage of Ramos S‐orange cells associated with THP‐1 monocytes (% association) was extracted.

### Data normalisation

The antibody MFI values of purified antibody binding (IgG and IgA) to the RBD single mutants (*n* = 13) were normalised to the maximum antibody binding (100%) for each variant to account for differences in coupling efficiency of the RBD mutants. The normalised purified antibody binding was used to calculate EC_50_s for purified IgG and IgA. EC_50_s > 400 were set to the threshold of 400.

### Data analysis

Traditional statistical analyses were performed with GraphPad Prism 9 (refer to Figure captions for details). Partial least squares regression analyses were conducted to determine multivariate relationships between immune features and continuous variables (e.g. ACE2 binding inhibition) using Matlab with the statistics and machine learning toolbox (Mathworks) and PLS_Toolbox (Eigenvector Research Incorporated, Wenatchee, USA).

## AUTHOR CONTRIBUTIONS

**Samantha K Davis:** Data curation; formal analysis; investigation; methodology; writing – original draft; writing – review and editing. **Kevin Selva:** Investigation; writing – review and editing. **Ester Lopez:** Methodology; writing – review and editing. **Ebene R Haycroft:** Methodology; writing – review and editing. **Wen Shi Lee:** Methodology; writing – review and editing. **Adam K Wheatley:** Resources; writing – review and editing. **Jennifer A Juno:** Investigation; writing – review and editing. **Amy Adair:** Resources; writing – review and editing. **Phillip Pymm:** Resources; writing – review and editing. **Samuel J Redmond:** Resources; writing – review and editing. **Nicholas A Gherardin:** Resources; writing – review and editing. **Dale I Godfrey:** Funding acquisition; resources; writing – review and editing. **Wai‐Hong Tham:** Funding acquisition; resources; writing – review and editing. **Stephen J Kent:** Conceptualization; funding acquisition; investigation; project administration; supervision; writing – review and editing. **Amy W Chung:** Conceptualization; formal analysis; funding acquisition; investigation; methodology; project administration; supervision; writing – original draft; writing – review and editing.

## Conflict of interest

NAG and DIG are coinventors on two provisional patents describing SARS‐CoV‐2 RBD vaccines and one provisional patent describing a diagnostic test for SARS‐CoV2 neutralising antibodies, submitted through the University of Melbourne Australia.

## Supporting information


**Supporting information S1** Supplementary material
