# Supplementary material for: Heterologous SARS‐CoV‐2 IgA neutralising antibody responses in convalescent plasma
Source: Clin Transl Immunology. 2022 Oct 23;11(10):e1424. doi: 10.1002/cti2.1424 (PMC9588388; doi:10.1002/cti2.1424)
Supplement: Supplementary file 1 — Supporting information S1 Supplementary material [file CTI2-11-0-s001.docx]

**
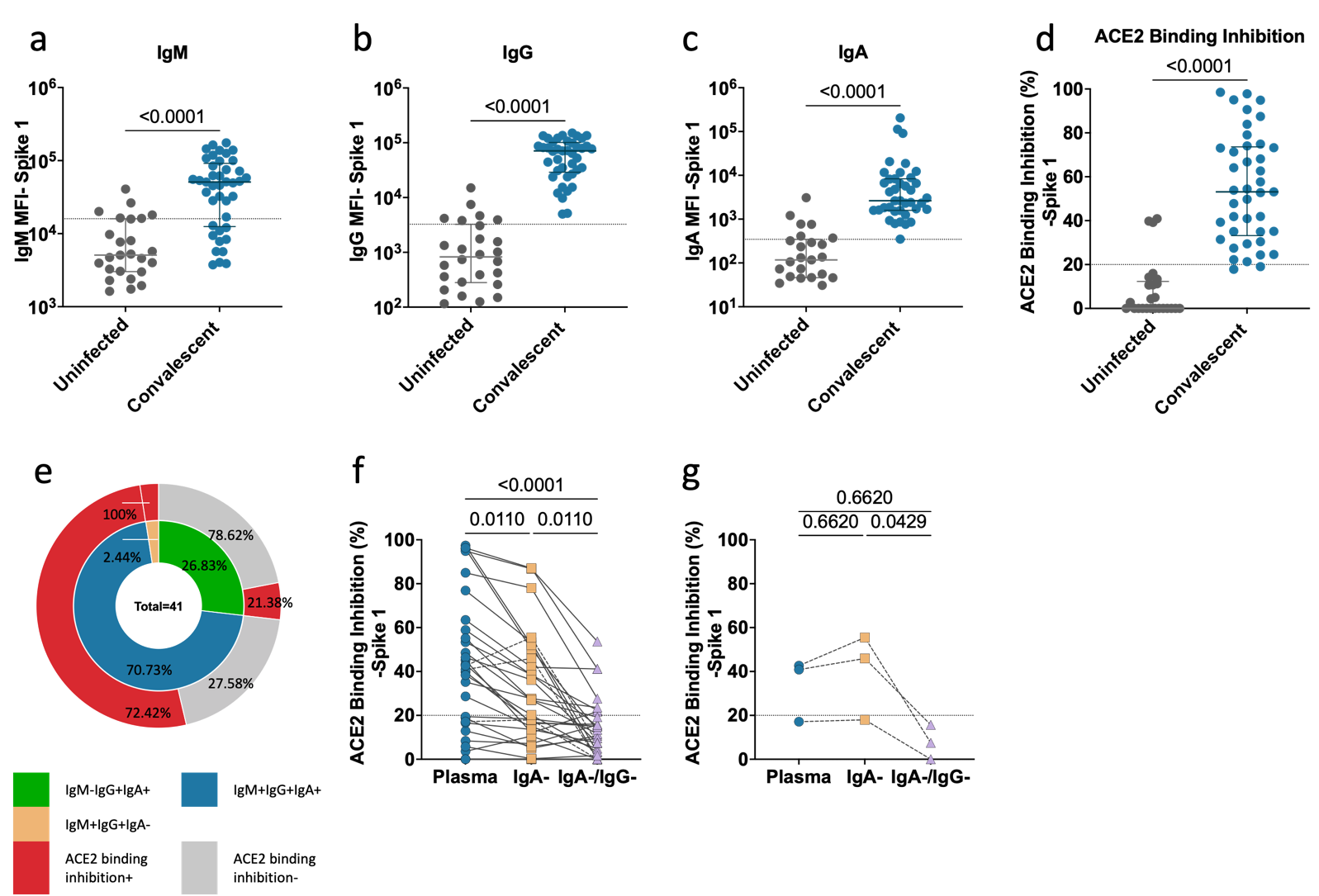
**

**Supplementary figure 1.** **Spike-1 antibody isotype binding and ACE2 binding inhibition (%) for plasma depleted plasma fractions and purified IgG and IgA**

IgM **(a)**, IgG **(b)** and IgA **(c)** antibody binding to SARS-CoV-2 spike-1 (S1) was assessed for convalescent (*n* = 41) (blue) and uninfected control (*n* = 26) (grey) plasma at a final dilution of 1:100 via multiplex. A positive threshold (grey dotted line) was defined as the 75^th^ percentile of antibody binding (MFI) for uninfected control plasma. **(d)** S1-ACE2 binding inhibition (%) of convalescent (blue) and uninfected control (grey) plasma (1:100 dilution). A positive threshold (grey dotted line) was defined as >20% ACE2 binding inhibition. **(e)** A pie chart outlining the percentage of subjects seropositive for anti-S1 antibody isotypes (IgM^-^IgG^+^IgA^+^ (green), IgM^+^IgG^+^IgA^+^ (blue), IgM^+^IgG^+^IgA^-^ (yellow)) (inner ring) and the percentage of each seropositive subset with ACE2 binding inhibition (red) (outer ring). **(f)** SARS-CoV-2 S1 ACE2 binding inhibition (%) of convalescent plasma (diluted 1:200; blue) and matched dilutions of IgA depleted (IgA^-^; yellow) and IgA and IgG depleted (IgA^-^/IgG^-^; purple) plasma fractions (*n* = 30). **(g)** A subset of samples where IgA depletion resulted in increased RBDWT-ACE2 binding inhibition (*n* = 3). Statistical analyses between two groups were performed using the Mann-Whitney *U*-test. Statistical analyses between plasma and depleted fractions were performed using the Friedman test with the Dunn’s multiple comparison test.

b

a

f

e

d

c

**Supplementary figure 2.** **Correlations of age, sex, and disease severity to Anti-SARS-CoV-2 IgA antibody binding**

Pearson R correlations of convalescent SARS-CoV-2 plasma (*n* = 41) IgA binding MFI to age for RBDwt **(a)**, spike trimer **(b)**, spike-2 (S2) **(c)** and spike- 1 (S1) **(d)** (median years = 55, IQR = 49-61). **(e)** Anti-RBDwt IgA binding (MFI) for male (*n* = 30) (blue, median MFI = 5191) and female (*n* = 11) (red, median MFI = 3347) subjects. **(f)** Anti-RBDwt IgA binding (MFI) for mild (*n* = 27) (blue, median MFI = 3596), moderate (*n* = 7) (red, median MFI = 9644) and severe disease (*n* = 2) (green, median MFI = 5817). 5 subjects had unknown disease severity and are not shown here.

**
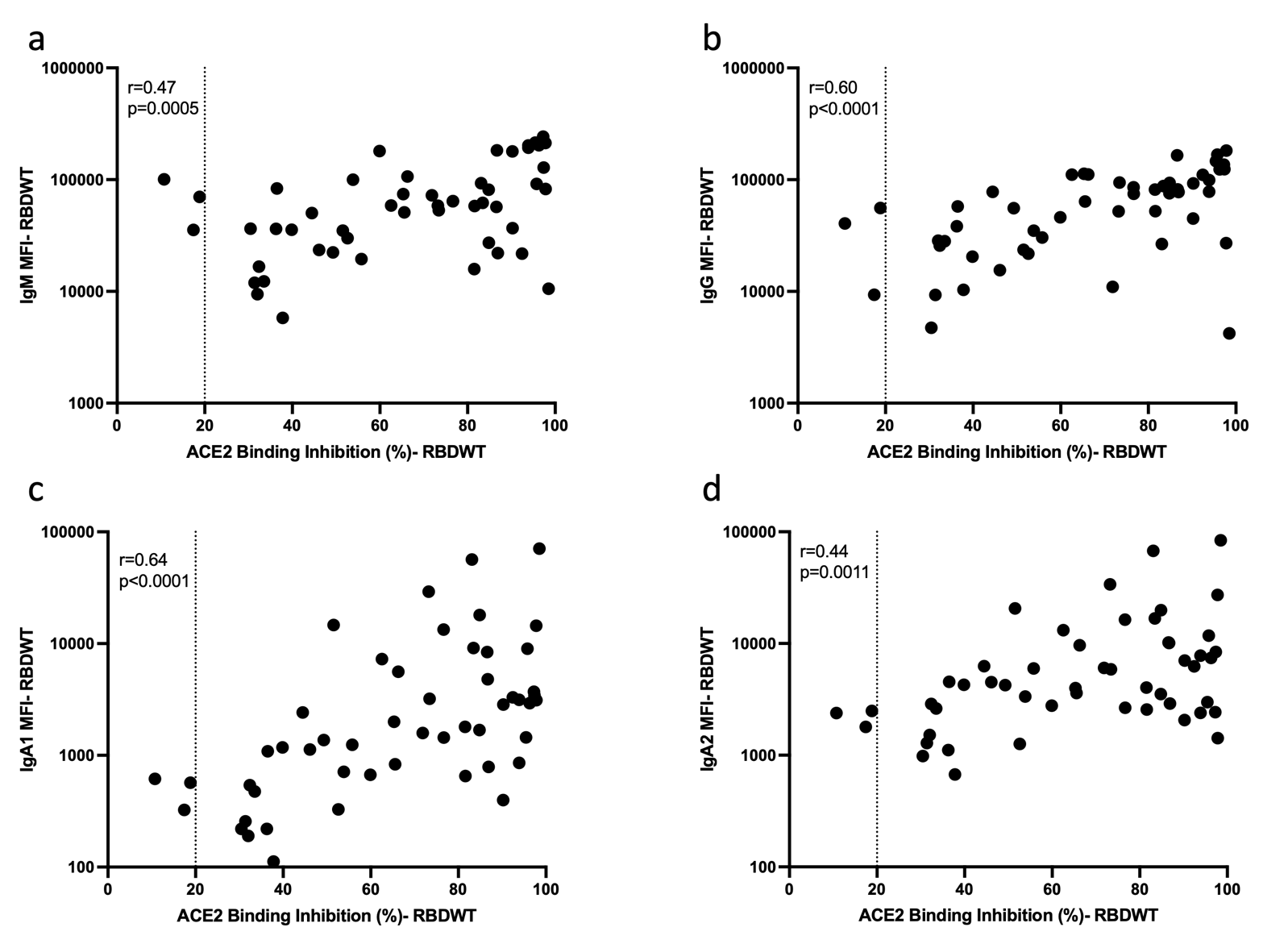
**

**Supplementary figure 3. Univariate correlations of convalescent plasma antibody binding to ACE2 binding inhibition (%) for RBDWT**

Non-parametric Spearman correlations between RBDWT antibody binding MFI and RBDWT-ACE2 binding inhibition (%) for plasma IgM **(a)**, IgG **(b)**, IgA1 **(c)** and IgA2 **(d)**. A positive threshold (grey dotted line) was defined as >20% ACE2 binding inhibition.


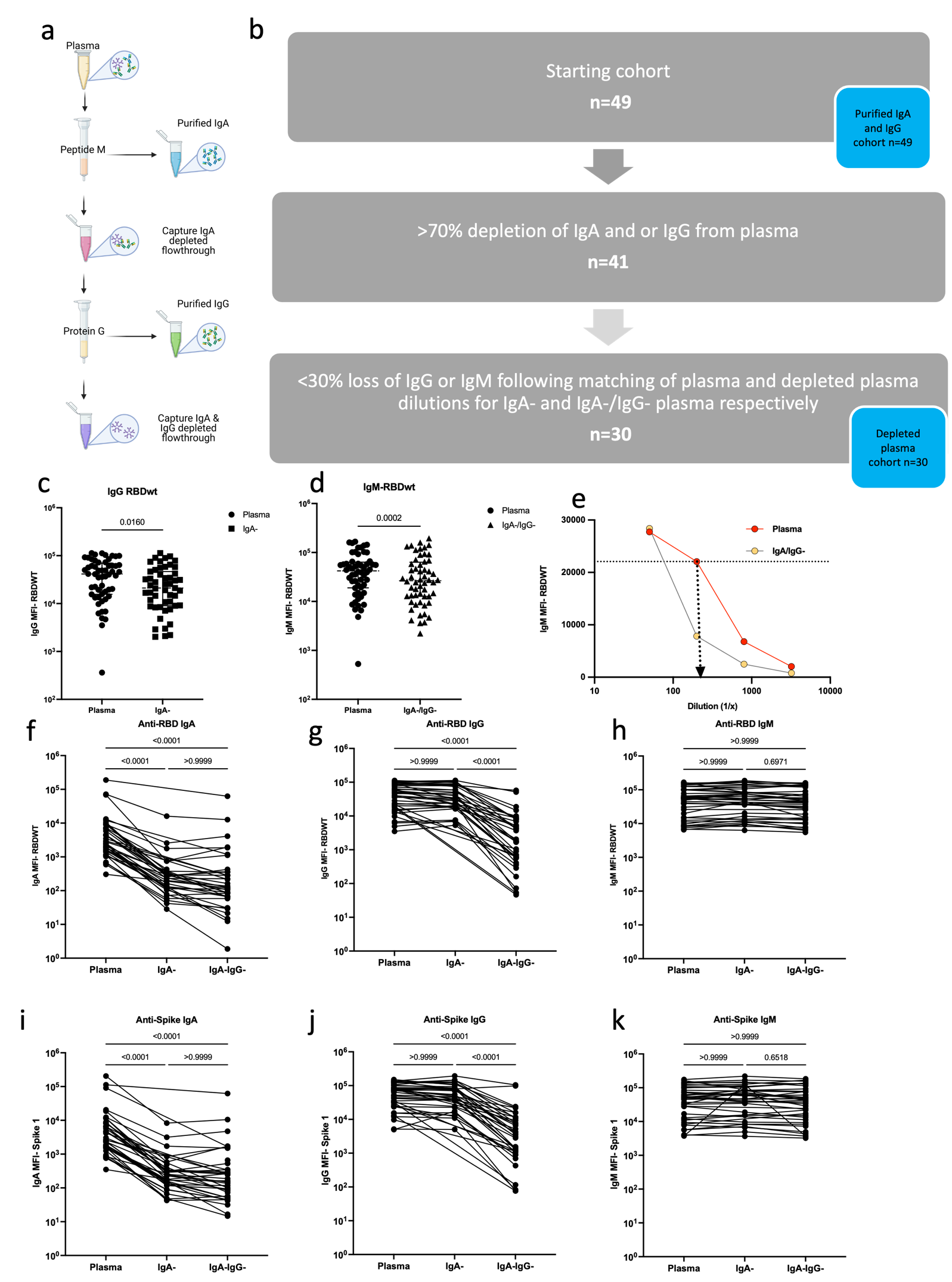


**Supplementary figure 4. Workflow of sample processing and quality control for IgG and IgA depletion**

Summary of the depletion and purification of IgA and IgG from convalescent plasma **(a)** and sample exclusion as defined in the methods **(b)**. A significant loss of RBDWT IgG **(c)** and IgM **(d)** was observed in IgA depleted (IgA^-^) and IgA and IgG depleted (IgA^-^/IgG^-^) plasma respectively (1:100 dilution) (*n* = 49). To ensure fair comparisons were made, depleted plasma dilutions were matched to whole plasma at a 1:100 dilution (*n* = 49). **(e)** Using RBDWT IgG or IgM binding MFI of IgA^-^ depleted and IgA^-^/IgG^-^ depleted plasma respectively, a plasma dilution was selected to match the antibody MFI of whole plasma (yellow) at 1:100 dilution (*n* = 49). Matching is indicated by the black dotted arrow. The RBDWT IgA **(f)**, IgG **(g)** and IgM **(h)** MFI for plasma, IgA^-^ depleted and IgA^-^/IgG^-^ depleted plasma for matched dilutions prior to sample exclusion (*n* = 49). The Spike-1 IgA **(i)**, IgG **(j)** and IgM **(k)** binding MFI for plasma, IgA^-^ depleted and IgA^-^/IgG^-^ depleted plasma for matched dilutions prior to sample exclusion (*n* = 49). See “Antibody depletion quality control and dilution matching” section for detailed methodology. Figure 4a created with Biorender.com.

d

c

b

a

h

g

f

e

**Supplementary figure 5. Purified convalescent IgG and IgA spike 1 and RBDWT supplementary data**

Purified IgA (blue) **(a)** and IgG (green) **(b)** spike-1 (S1) antibody binding (MFI) for uninfected (*n* = 6, grey) and convalescent (*n* = 49) plasma at 100 μg mL ^-1^ total antibody. **(c)** Purified IgA (blue) and IgG (green) S1-ACE2 binding inhibition (%) at 100 μg mL ^-1^ total antibody (*n* = 49). Purified IgA (blue) **(d)** and IgG (green) **(e)** RBDWT antibody binding (MFI) for uninfected (*n* = 6, grey) and convalescent (*n* = 49) subjects at 100 μg mL ^-1^ total antibody. Purified IgA (blue) **(f)** and IgG (green) **(g)** RBDWT-ACE2 binding inhibition for uninfected (*n* = 6, grey) and convalescent (*n* = 49) cohorts at 100 μg mL ^-1^ total antibody. **(h)** A comparison of purified IgA (blue) and IgG (green) RBDWT-ACE2 binding inhibition (%) at 100 μg mL ^-1^ total antibody (*n* = 49). Lines connect IgG and IgA for a single individual. A positive threshold (grey dotted line) was defined as >20% ACE2 binding inhibition.

**
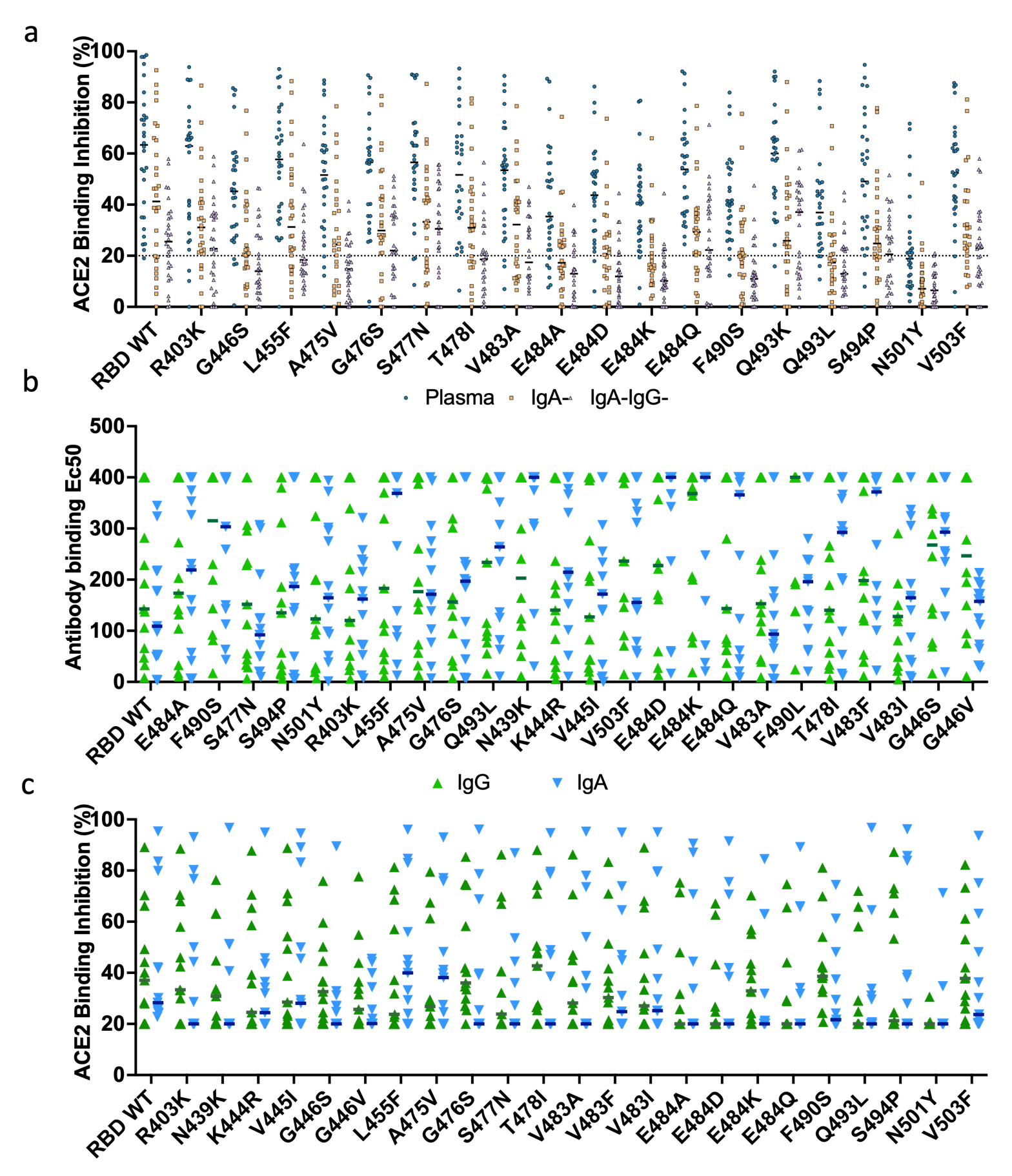
Supplementary figure 6. Raw ACE2 binding inhibition and antibody binding data for depleted plasma and purified antibodies to single RBD mutations**

**(a)** The ACE2 binding inhibition (%) to RBDWT and 18 other RBD single mutants for plasma (blue), IgA depleted (IgA^-^, yellow) and IgA and IgG depleted plasma (IgA^-^IgG^-^, purple triangle). The raw purified IgG (green) and IgA (blue) Ec50’s **(b)** and ACE2 binding inhibition (%) **(c)** for RBDWT and 23 other RBD single mutants. Lines depict the median response for each sample type to each RBD.

**
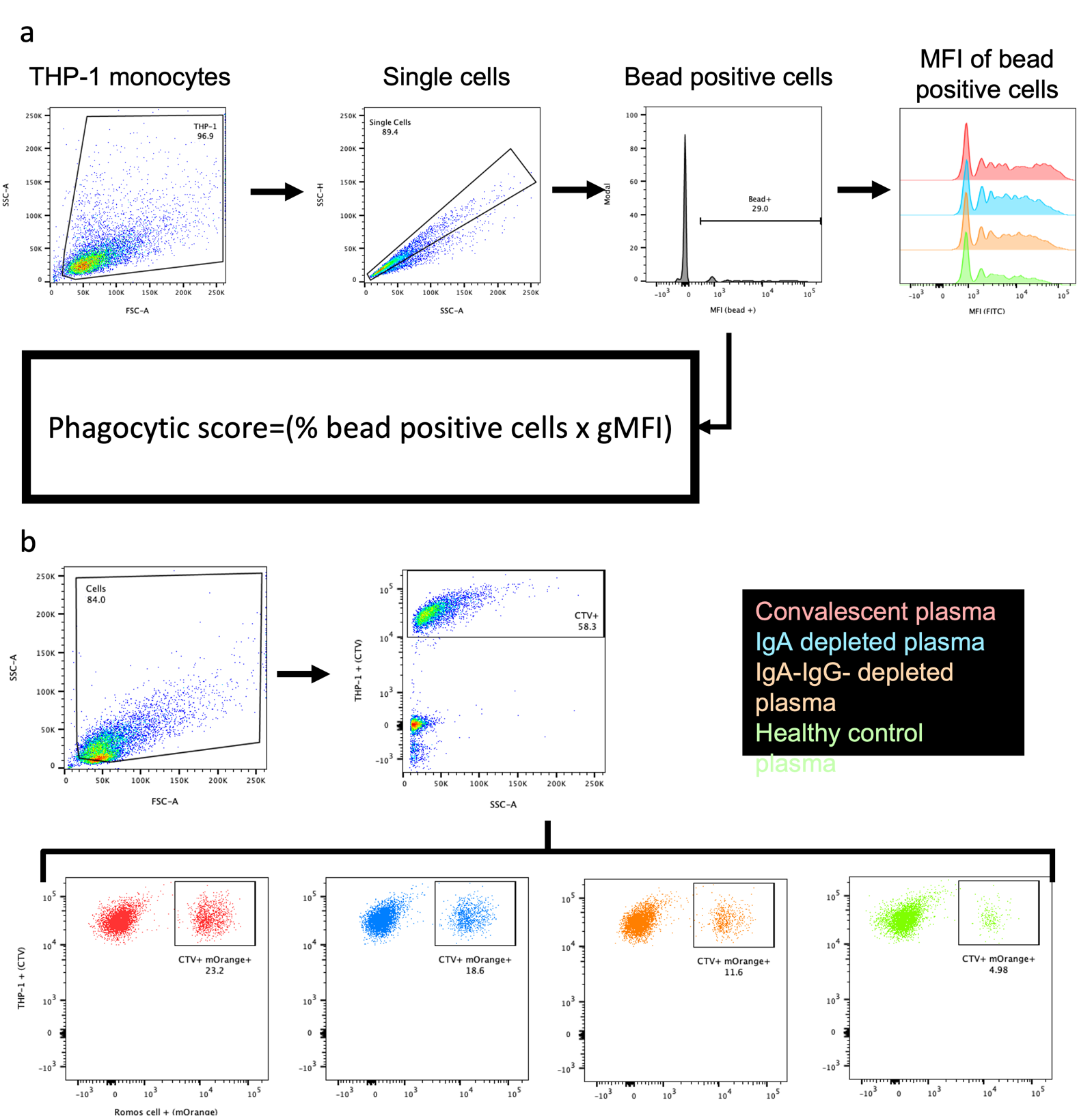
Supplementary figure 7. Gating strategy of ADP bead-based assay and THP-1 Ramos S-orange association assay**

**(a)** The antibody dependent bead-based phagocytosis assay (ADP assay) gating strategy. First, THP-1 monocytes were gated, followed by single cells and bead positive cells. Finally, a phagocytic score was calculated. **(b)** The THP-1 and Ramos S-orange cell association assay gating strategy. Cells were gated, followed by cell trace violet (CTV) stained THP-1 monocytes. Finally, CTV^+^ (THP-1) mOrange^+^ (Ramos S-orange) cells were gated, and the percentage of double positives was recorded as a measure of anti-spike antibody mediated cell association. Example gating for convalescent plasma (red), IgA^-^ depleted plasma (blue), IgA^-^/IgG^-^ depleted plasma (orange) and healthy control plasma (green) are shown.

**Supplementary table 1. Summary of cohort demographics**

|  | | **Healthy controls** | | **COVID-19 Convalescent** | |
| --- | --- | --- | --- | --- | --- |
|  |  | **Multiplex (n=26)** | **Functional (n=12)*** | **Multiplex (n=41)** | **Functional (n=39)*** |
| **Sample collection data (month)** | | **April-May 2020** | **April-May 2020** | **April-May 2020** | **April-May 2020** |
| **Date of PCR test (month)** | |  |  | **March 2020** | **March 2020** |
| **Age, Median (IQR)** | | **54 (24-60)** | **55 (24-63)** | **55 (49-61)** | **57 (52-69)** |
| **Gender, female (%)** |  | **12 (48)** | **5 (45.45)** | **11 (26.83)** | **11 (28.21)** |
| **PCR and/or serology** |  | **Negative**** | **Negative**** | **Positive** | **Positive** |
| **Disease Severity (self-report)** | **Mild (%)** | **-** | **-** | **27 (65.85)** | **26 (68.42)** |
|  | **Moderate (%)** | **-** | **-** | **7 (17.07)** | **6 (15.79)** |
|  | **Sever (%)** | **-** | **-** | **2 (4.88)** | **1 (2.63)** |
|  | **No details (%)** | **-** | **-** | **5 (12.20)** | **5 (13.16)** |
| **Days post positive COVID test, median (IQR)** | | **-** | **-** | **36 (30-44)** | **36 (30-38)** |
| **Days post symptom onset, median (IQR)** | | **-** | **-** | **41 (36-47)** | **40 (38-42)** |

- Not applicable

*Individuals were randomly selected from original cohort

**Confirmed negative using serology

**Supplementary table 2. Matched depleted plasma dilution to whole plasma at 1:100 dilution**

|  | Subject ID | Dilution IgA^-^ (1/n) | Dilution IgA^-^/IgG^-^ (1/n) |
| --- | --- | --- | --- |
| 1 | CP07 | 100 | 100 |
| 2 | CP11 | 50 | 50 |
| 3 | CP15 | 50 | 50 |
| 4 | CP19 | 100 | 100 |
| 5 | CP23 | 100 | 100 |
| 6 | CP25 | 100 | 100 |
| 7 | CP26 | 100 | 100 |
| 8 | CP27 | 50 | 50 |
| 9 | CP29 | 100 | 100 |
| 10 | CP30 | 100 | 50 |
| 11 | CP31 | 50 | 50 |
| 12 | CP32 | 50 | 100 |
| 13 | CP35 | 100 | 50 |
| 14 | CP39 | 100 | 100 |
| 15 | CP43 | 100 | 100 |
| 16 | CP44 | 100 | 100 |
| 17 | CP45 | 100 | 100 |
| 18 | CP46 | 100 | 100 |
| 19 | CP51 | 100 | 100 |
| 20 | CP52 | 50 | 50 |
| 21 | CP53 | 50 | 50 |
| 22 | CP54 | 50 | 100 |
| 23 | CP57 | 100 | 100 |
| 24 | CP58 | 50 | 100 |
| 25 | CP60 | 100 | 100 |
| 26 | CP61 | 100 | 100 |
| 27 | CP63 | 100 | 100 |
| 28 | CP66 | 50 | 50 |
| 29 | CP68 | 50 | 100 |
| 30 | CP70 | 50 | 50 |
